# Development of a semi-automated segmentation tool for high frequency ultrasound image analysis of mouse echocardiograms

**DOI:** 10.1038/s41598-021-85971-3

**Published:** 2021-03-22

**Authors:** Kristi Powers, Raymond Chang, Justin Torello, Rhonda Silva, Yannick Cadoret, William Cupelo, Lori Morton, Michael Dunn

**Affiliations:** 1 Regeneron Pharmaceuticals, 777 Old Saw Mill River Road, Tarrytown, NY 10591 USA; 2 Invicro LLC, Dry Dock Road, Boston, MA USA

**Keywords:** High-throughput screening, Imaging, Software, Drug discovery, Molecular biology, Physiology, Structural biology, Systems biology, Cardiology, Medical research, Scientific data, Software

## Abstract

Echocardiography is a widely used and clinically translatable imaging modality for the evaluation of cardiac structure and function in preclinical drug discovery and development. Echocardiograms are among the first in vivo diagnostic tools utilized to evaluate the heart due to its relatively low cost, high throughput acquisition, and non-invasive nature; however lengthy manual image analysis, intra- and inter-operator variability, and subjective image analysis presents a challenge for reproducible data generation in preclinical research. To combat the image-processing bottleneck and address both variability and reproducibly challenges, we developed a semi-automated analysis algorithm workflow to analyze long- and short-axis murine left ventricle (LV) ultrasound images. The long-axis B-mode algorithm executes a script protocol that is trained using a reference library of 322 manually segmented LV ultrasound images. The short-axis script was engineered to analyze M-mode ultrasound images in a semi-automated fashion using a pixel intensity evaluation approach, allowing analysts to place two seed-points to triangulate the local maxima of LV wall boundary annotations. Blinded operator evaluation of the semi-automated analysis tool was performed and compared to the current manual segmentation methodology for testing inter- and intra-operator reproducibility at baseline and after a pharmacologic challenge. Comparisons between manual and semi-automatic derivation of LV ejection fraction resulted in a relative difference of 1% for long-axis (B-mode) images and 2.7% for short-axis (M-mode) images. Our semi-automatic workflow approach reduces image analysis time and subjective bias, as well as decreases inter- and intra-operator variability, thereby enhancing throughput and improving data quality for pre-clinical in vivo studies that incorporate cardiac structure and function endpoints.

## Introduction

Echocardiography is a non-invasive, cost effective and reliable imaging modality used extensively for the clinical assessment of cardiovascular function and medical diagnosis^1,2^. In addition to its clinical utility, echocardiography is widely used in preclinical basic and applied research for the evaluation of myocardial structure and function in the drug discovery and development arenas^3^. Applications in normal and diseased preclinical animal models have gained a significant foothold in both drug development and academic research^4^. With the advent of high frequency micro-ultrasound systems, (in the order of 10 to 40 MHz frequency transducers) routine assessment of cardiac structure and function of murine models has proven valuable in interrogating the pathophysiology associated with cardiovascular diseases, allowing for enhanced understanding of mechanisms of action that occur in disease processes^5,6^.

The appeal surrounding preclinical micro-ultrasound as a technology in the drug development space stems from its relative affordability, widespread availability, translatability and rapid real-time image acquisition supporting high throughput imaging^4^. Nevertheless, the technology presents with challenges that include requiring significant technical expertise for data acquisition and analysis. This complexity contributes to intra- and inter-operator and between-institution variability that impacts data interpretation. Moreover, analysis of echocardiographic data is highly subjective and creates a lengthy manual image analysis process that results in a workflow bottleneck^7^. Internal audit of time required from murine echocardiography acquisition to complete data analysis and peer review shows that it requires a minimum of thirty minutes per mouse. This limitation surrounding ultrasound analysis throughput is amplified at pharmaceutical/biotechnology companies and contract research organizations where analysis of large sample sizes routinely creates a data bottleneck and results in the slowing down of research efforts aimed at developing novel therapeutics.

Although routinely used clinically, the acquisition and analysis of human echocardiograms is typically not conducted at the scale performed in the drug development space. Despite lower overall throughput, clinical image analysis has seen recent and substantial advancements in quantitative ultrasound image analysis with the development of model-based 2D echocardiographic image tracking^8^. When comparing the clinical advances to the current state of preclinical micro-ultrasound image analysis, the discrepancy leaves an unmet need to innovate methods that enhance data processing in a high throughput fashion, permitting rapid interpretation of data and quick decision-making.

To address this need, our goal was to develop a software-based analysis algorithm for the semi-automated segmentation of murine left ventricle (LV) long- and short-axis ultrasound images. Described herein, a semi-automated method for LV segmentation and derivation of volumetric and functional parameters for mice was developed. Our micro-ultrasound analysis tool performs favorably relative to a manual analysis approach, and reduces the time required for image analysis. In addition, we demonstrate that our tool reduces subjective bias as well as inter- and intra-operator variability, the main current limitations of ultrasound image analysis.

## Methods

All animal procedures and protocols described in this work were approved by Regeneron Pharmaceuticals, are carried out in compliance with ARRIVE guidelines, in accordance with state and federal guidelines and aligned with regulations set forth by Regeneron Pharmaceutical’s Institutional Animal Care and Use Committee.

### Animals and materials

Male C57BL/6N mice (n = 6), aged ~ 20 weeks and weighing 27.2–31.1 g (Charles River Laboratories, Wilmington, MA, 01887) were acclimated for a minimum of 7 days prior to experimentation. Mice were co-housed in polycarbonate, solid-bottom cages in a temperature-controlled environment (22 ± 2 °C), an approximate 12-h light–dark cycle, with access to research diets standard pellet chow and reverse osmosis filtered water. Isoproterenol hydrochloride powder was purchased from Sigma Aldrich and was diluted with saline to achieve an injected dose concentration of 5 mg/kg (100 mg; lot#: SLBC2168V; St. Louis, MO).

### In vivo imaging procedure

Each mouse was placed inside a warm (~ 37 °C) induction chamber (warmed by electrical blanket and/or a warm water circulating blanket) containing a gas mixture of 2% isoflurane in 100% oxygen for 2 to 3 min. Upon confirmation of appropriate anesthetic plane via a negative toe or tail pinch, the chest area (from the collarbone to slightly below the diaphragm) was shaved and depilatory cream (Nair) was applied to ensure that any hair in the imaging region of interest was removed. The animal was then transferred to the animal imaging platform, positioned supine, and their paws were secured with medical tape to gold plated ECG leads coated with conduction cream. A rectal probe was then inserted and taped down to monitor the animals’ body temperature during the time of acquisition. All scans were conducted on mice that had a targeted core body temperature of 37 ± 1 °C and a heart rate of 400–500 beats per minute.

Mouse left ventricle echocardiograms were acquired using the Vevo2100 (Visualsonics/FujiFilm, Toronto, Canada) high frequency ultrasound machine and the 18–38 MHz transducer (MS400). Images were acquired on a warmed (37.5 °C) imaging platform, oriented to approximately 40°–50° so that the lower left-hand corner of the platform was pulled down. Utilizing a probe holder, the transducer was pivoted outward roughly 10°–15° toward the right side of each animal, ensuring that the scanning head would be placed parallel to the axis of the left ventricle and the sternum. Ultrasonic transmission gel was placed on the chest of the animal to obtain a parasternal long axis (PLAX) B-mode cine loop ensuring a clear image containing both the apex and aortic valve. A cine loop of 300 frames (6.67 ms/per frame) was acquired and the transducer was then rotated clockwise 90°. The M-mode curser was placed in the center of the left ventricle chamber to acquire a 5-second cine-loop short axis (SAX) M-mode image at the level of the papillary muscle as a landmark for reproducible slice location.

### Reference library for parasternal long axis B-mode analysis

The underlying concept behind the development of the semi-automated segmentation algorithm for analysis of PLAX B-mode ultrasound images was derived from the multi-atlas or reference library approach used in clinical ECG wave pattern recognition analysis^9^. Following cine loop acquisition, images were exported and stored on a data server for import into both the Visualsonics analysis software suite (Version 3.1.1) and the Image Study Data Management Software Platform (ISDMP) (iPACS-a research PACS [picture archival and communication system] Invicro, LLC/Konica Minolta, Boston. MA). PLAX B-mode ultrasound images containing a distribution of both healthy and various murine models of cardiovascular disease were manually traced by highly trained and validated sonographers (within analyst coefficient of variation at or below 10–20%—data not shown) as well as put through an internal blinded peer review process. This specific workflow ensured a high level of analysis accuracy and agreement before images were incorporated into the reference library for the computer learning development of the semi-automated segmentation algorithm. Exploiting the presence of characteristic anatomical patterns seen in LV ultrasound images functioned to train the computer algorithm to recognize these patterns within unanalyzed or newly acquired ultrasound images^10^. To obtain functional, clinically relevant endpoints, the left ventricle B-mode images were traced at end-diastole and end-systole. An adequate number of points were manually placed along the endocardial wall of the left ventricle at each phase to capture an entire cardiac cycle, with accurate tracking between phases. A package of manually analyzed murine echocardiograms containing a range of images of baseline scans, post-pharmacologic challenge, post-surgically induced disease state models, and general genetic phenotyping models were aggregated to create a working library. This library contained roughly 322 manually traced mouse PLAX ultrasound images from transgenic mice of mixed C57BL/6 and 129SV genetic background (ranging from 75 to 87% C57BL/6). The analyzed images contained a broad selection of echocardiograms representative of the expected variability often seen in animal models.

### Creation of semi-automated segmentation algorithm for LV long-axis B-mode and short-axis M-mode echocardiograms

For B-mode segmentation, the VivoQuant Whole-body Multi-Atlas Segmentation tool (Invicro, LLC/Konica Minolta, Boston, MA) was used with the reference library described above^10^. Following denoising (including image down-sampling by a factor of 2), for each systolic and diastolic frame, the manually traced region of interest (ROI) of the reference library were affinely-registered to the selected image with normalized mutual information (NMI) as the objective function. Following co-registration, the labels of the five registrations with the highest NMI were mapped and averaged to create a probability map (based on pixel-by-pixel mapping of images) that a threshold was then applied to generate the final segmentation used for analysis. Prior to overall deployment, the method described above was tested and validated on a subset of echocardiograms acquired from control and post-surgically induced disease mouse models containing a total of 90 images (30 per model). For each set of echocardiograms, a working reference library was created from approximately 25 randomly selected images amongst the group of interest, with the remaining 5 images defined as test subjects. Following analysis using the developed tool, the resulting test subject segmentations were compared to manual peer reviewed segmentations performed by blinded independent operators. Dice coefficients from comparisons of semi-automated and manual segmentations ranged from 0.85 to 0.98 with an average overlap ratio of 0.93 ± 0.03 (data not shown).

The short axis M-mode algorithm works in a fashion similar to the concept of statistical parametric mapping used to evaluate differences within brain activity during functional neuroimaging experiments^11^. Instead of assessing images on a voxel-by-voxel basis, the algorithm works by evaluating 2D echocardiography in a pixel-by-pixel manor. More specifically, it works by using a two seed-point approach to triangulate the local maxima of the anterior and posterior endocardium and epicardium, from which it automatically populates a best fit line for each. The M-mode segmentation algorithm is based on user selection of two points that define a search region around two walls of interest. The steps required for the manual selection of the search regions are further described in section “Semi-automated segmentation tool workflow implementation” Once two points have been selected, noise reduction and gradient filtering are applied to the selected region. A gradient-based cost function is applied to every pixel of the region to derive a cost function image. The cost function image is used as the input to a dynamic programming routine that computes an optimal path, thus defining the wall boundary in the defined region^12^. The same process is repeated for each of the four walls (8 seed points). After all the walls have been processed, each segmentation is filtered in the spectral domain to keep only the highest frequency. The peaks of the filtered signal are used across the four walls to compute the wall measurements over systolic and diastolic sections (including wall-thicknesses and functional dimensions). In addition to performing left ventricle segmentation and volumetric analysis, the semi-automated segmentation algorithm includes automatic calculation of clinically relevant parameters used to assess left ventricle structure and function in preclinical species incorporated from both PLAX B-mode and SAX M-mode images.

### Validation study

A pilot study was conducted to test the validity and reproducibility of the semi-automated segmentation algorithm within a routine drug development workflow. Echocardiograms were acquired from C57BL/6 mice (n = 6) at baseline and again fifteen minutes after a single 5 mg/kg subcutaneous dose of the beta agonist, isoproterenol, (Isoproterenol hydrochloride powder; 100 mg; Sigma Aldrich; lot#: SLBC2168V). Each individual mouse was considered the experimental unit and served as its own control. The investigator assisting the sonographer was the only one aware of the treatment group allocation. The sample size of six per group was selected to ensure adequately powered statistical analyses while minimizing the total number of animals. No animals were excluded from analysis. Following acquisition, the images were manually analyzed via the Vevolabs analysis software (Visualsonics/FujiFilm, Toronto, Canada) by three independent, skilled and blinded operators using the protocol previously described. The same images were then analyzed by the same operators using the semi-automated segmentation algorithm. Bi-variate regression analyses of manual and semi-automated segmented images were performed to assess variation between sonographers using the tool. To assess the level of variation with a single sonographer using the semi-automated segmentation tool, a coefficient of variation analysis was performed by running the semi-automated segmentation tool in triplicate fashion on the same data set. This protocol was followed to assess the variation of both B-mode and M-mode imaging segmentations. Similar coefficient of variation analysis was also performed on manually traced ultrasound images in the same triplicate fashion for a head-to-head comparison with the algorithm. Objectivity was achieved during the coefficient of variation quantification for the semi-automatic segmentation tool by ensuring that images were devoid of any peer review processes. Removal of the peer review process was essential to mimic the procedures of the manual coefficient of variation analysis for a proper comparison.

### Semi-automated segmentation tool workflow implementation

The algorithm was developed and deployed to Regeneron pharmaceuticals via a commercial image viewing and analysis software^10^. This software provides a graphical user interface-driven workflow tool for incorporation of the semi-automated analysis algorithms, with the added capability of integrating into a central ISDMP. Once echocardiograms are acquired, they can be stored into the ISDMP using a data transfer application from source to the ISDMP content store^13^. Image data were then managed by the ISDMP and made available within the analysis software for execution of the semi-automated algorithm workflows.

### Data transfer and storage

An Invicro developed rsync wrapper desktop application, iPACSSync, automatically copies data from the Vevo2100 scanner workstation to a study specific repository in the iPACS. Once the sync is complete, designated users with the appropriate permissions can access the data on any computer through a web browser, without the need to download it locally. Within the iPACS, ultrasound data are viewed in a Windows-like directory file structure, or in a database-like view that displays important metadata such as subject identification, acquisition time, image type, etc.

### Segmentation and analysis

The post-processing suite can flexibly utilize a proprietary JavaScript-based programming language, Vivoscript, that can execute various tasks by leveraging the ISDMP in an automated fashion. The created analysis workflow enables open communication between the post-processing suite and data management system, allowing for users to batch process data and automatically store results back to the repository. Separate scripts for each imaging orientation were created. Each script executes the imaging mode-specific processing pipeline described in the following sections. The processing script executes each analysis protocol individually based off the different concepts related to each imaging mode. Each script executes the following:

#### For B-mode

- Queries the iPACS for data to process using user-defined study and patient filters;
- Downloads the queried images as raw data (locates PLAX images by identifying “B-mode” in the header file);
- Launches the VivoQuant B-mode importing tool;
- Prompts user to first select the frame at which the heart is in end-systole, followed by selecting the frame at which the heart is in end-diastole;
- Imports each selected frame as 2D images in VivoQuant and uploads them in a different study repository on the iPACS;
- Repeats this step for all animals in the study;
- Automatically starts running the selected 2D frames through the Multi-Atlas Segmentation tool in VivoQuant using the manually segmented reference library and a set of fixed parameters;
- Automatically generates a region of best fit;
- Automatically calculates functional measures of ejection fraction, stroke volume, fractional shortening, fractional area change, end-diastolic volume, end-systolic volume, end-diastolic area, end-systolic area, end-diastolic dimension, and end-systolic dimension;
- Submits the generated region of interest, screenshots of the segmentations, and measurements to the appropriate study repository on the iPACS.

#### For M-mode

**Figure 1:**
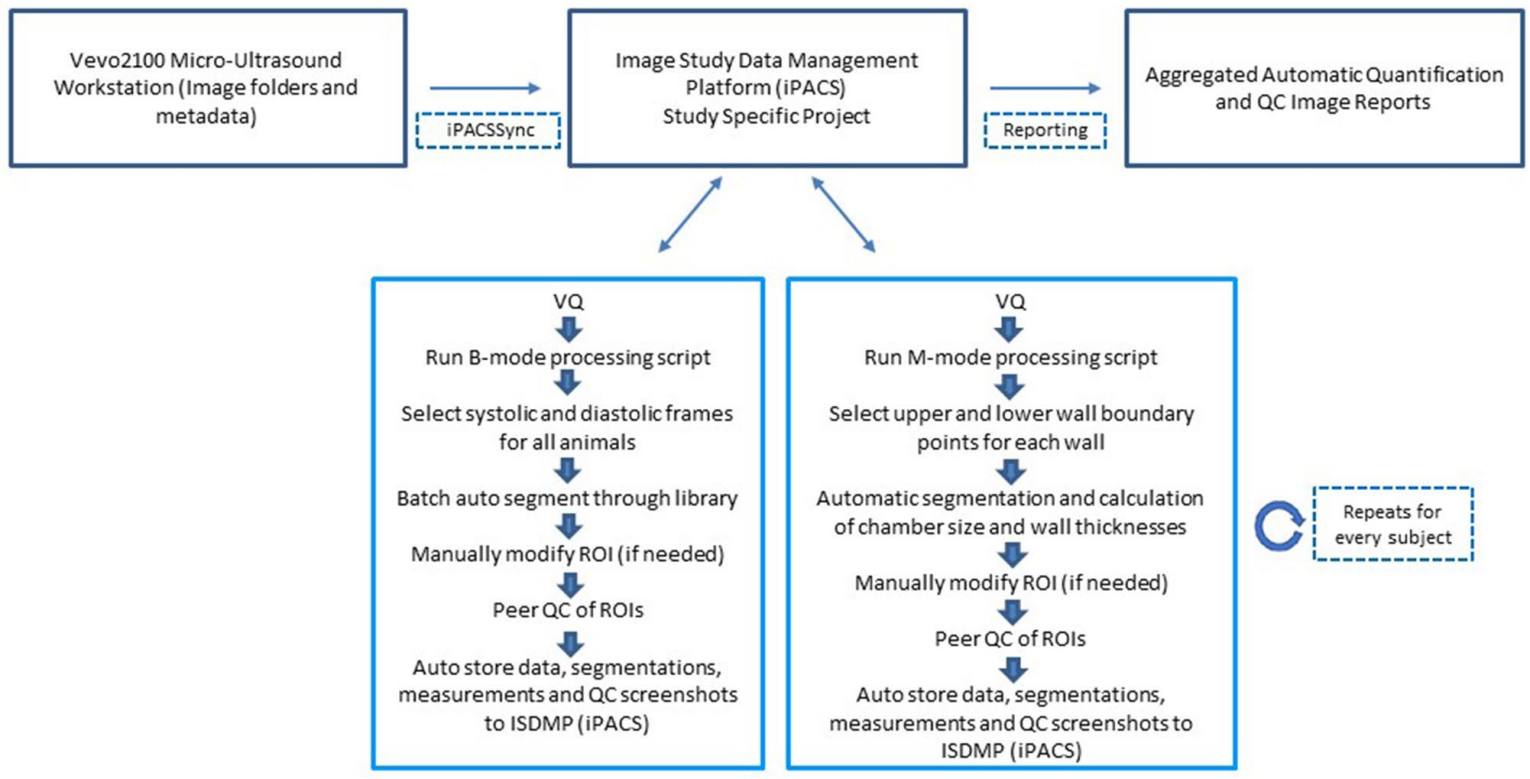
Semi-automated segmentation tool flowchart: pictorial representation of the semi-automated segmentation workflow for analyzing B-mode and M-mode echocardiograms.

- Queries the iPACS for data to process using user-defined study and patient filters;
- Downloads the queried images as raw data;
- Launches the VivoQuant M-mode importing tool;
- Automatically imports a pre-defined portion of the scan as new data in VivoQuant and uploads it in a different study repository on the iPACS;
- Launches the VivoQuant M-mode processing tool;
- Pauses to allow users to select upper and lower boundary points of each of the four walls;
- Automatically segments the four walls;
- Automatically calculates and displays LV inner diameter at systole, LV inner diameter at diastole, end systolic volume, end diastolic volume, stroke volume, ejection fraction, fractional shortening, LV mass, LV mass corrected to body surface area, LV anterior wall dimension at systole, LV anterior wall dimension at diastole, LV posterior wall dimension at systole and LV posterior wall dimension at diastole;
- Pauses to allow users to make any manual modifications to the segmentations and updates the measurements real time;
- Submits the processed data, screenshots of the segmentations, and measurements to the appropriate study repository on the iPACS;
- Repeats steps (b) through (i) for all data identified in (a) (Fig. 1).

### Results, aggregation and reporting

After analyzing all PLAX B-mode and SAX M-mode images in a study, users then navigate to the iPACS, select the data of interest, and generate reports. Two reports are available with each processing module, an excel file that contains quantification data and a QC PowerPoint with segmentation images for each subject. After reviewing the segmentations and output measures, users can load data into VivoQuant and make further changes or adjustments if neccessary. Updates to the segmentations at any point in time automatically update the reports so that only the most recently analyzed data are reviewed.

### Statistical analysis

The focus of the present analyses was largely centered around the goodness of fit of two different methods of image segmentation (algorithm-based versus manual-based) and three different ultrasound analysts (Analyst 1, Analyst 2, and Analyst 3). Bivariate linear regressions were made on the combinations between segmentation methods and different analysts. Coefficients of determination (*r*^2^), a measure of proportional accountability or goodness of fit between two measures were derived from Pearson’s regression analyses. Statistical significance for both *r* and *r*^2^ was set at an alpha (*p*) of less than or equal to 0.05 (*p* ≤ 0.05).

To determine reliability of image segmentation methods, coefficients of variation (mean/standard deviation) of the segmentation methods were calculated based on image segmentations performed in triplicate. For both assessments, a proper peer review and post-review image analysis processing were excluded to facilitate assessment of the performance of the algorithm alone. The resultant outcomes of the segmentations were used to calculate the coefficients of variation. To simplify the analyses of the coefficients of variation, student’s within-subjects *t*-tests were performed on the calculated coefficients of variation. Statistical significance was set at an alpha (*p*) of less than or equal to 0.05 (*p* ≤ 0.05). Our alternative hypothesis (*H*_*1*_) predicted that the coefficients of variation should not be different between methods and treatment conditions (saline versus isoproterenol). We also determined that a reliable segmentation outcome should produce coefficients of variation less than or equal to 20% of the standard deviation (≤ 20%). All statistical analyses were performed using Prism—GraphPad (version 7.0) and Microsoft Excel.

## Results

### B-mode segmentation

**Figure 2:**
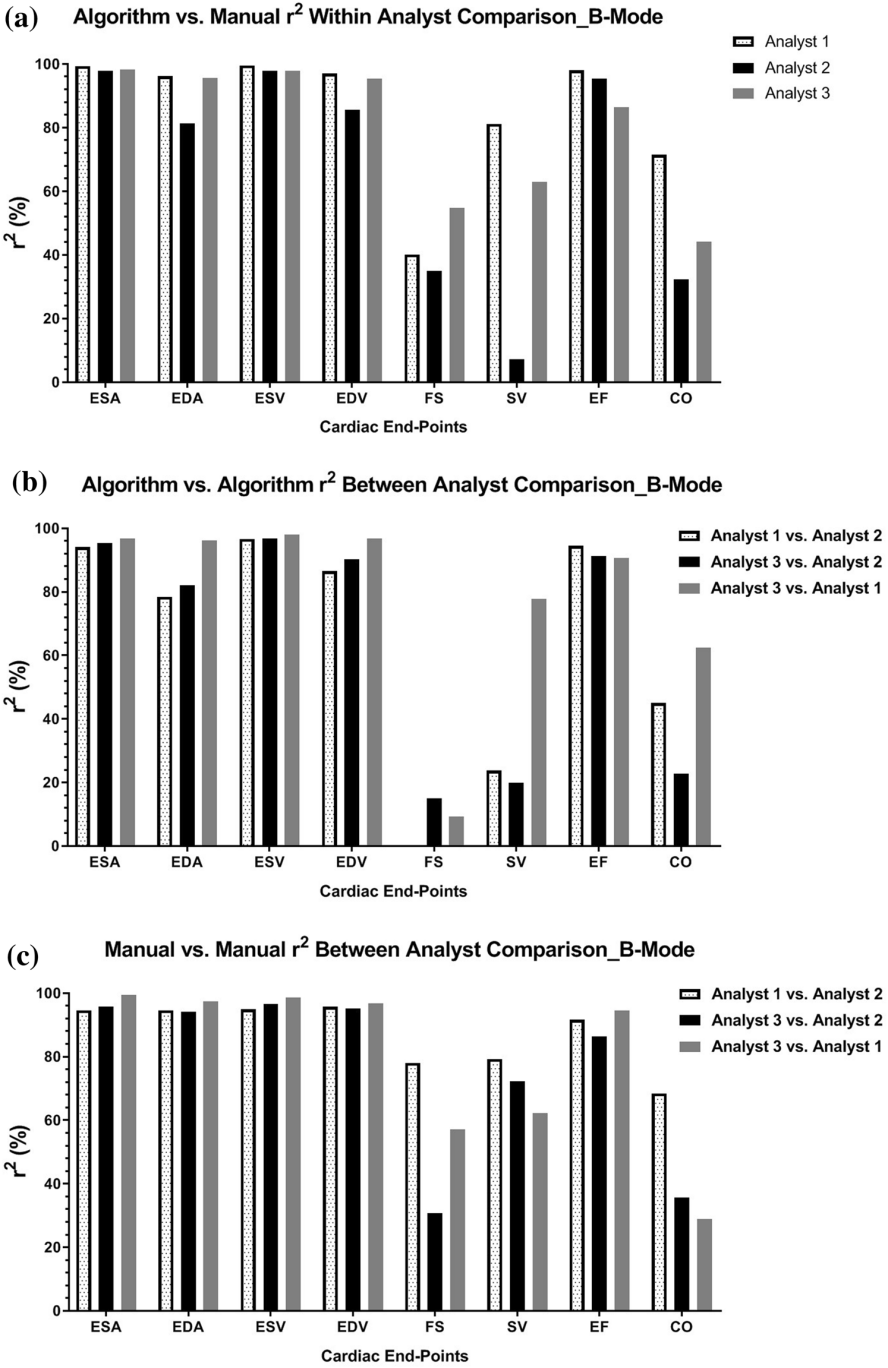
B-mode Segmentation Comparisons: Graphical representations of B-mode coefficient of determination (r^2^) among three analysts, with r^2^ values on the y-axis, and long axis LV structure and function end-points on the x-axis. (**a**) Displays the within analyst comparison of left ventricle B-mode echocardiograms segmented using the semi-automated algorithm compared to manual segmentation; (**b**) shows the between analyst comparison while employing the algorithm for segmentation; and (**c**) shows the between analyst comparison when manual segmentation is performed; p values set at p < 0.05.

**Figure 3:**
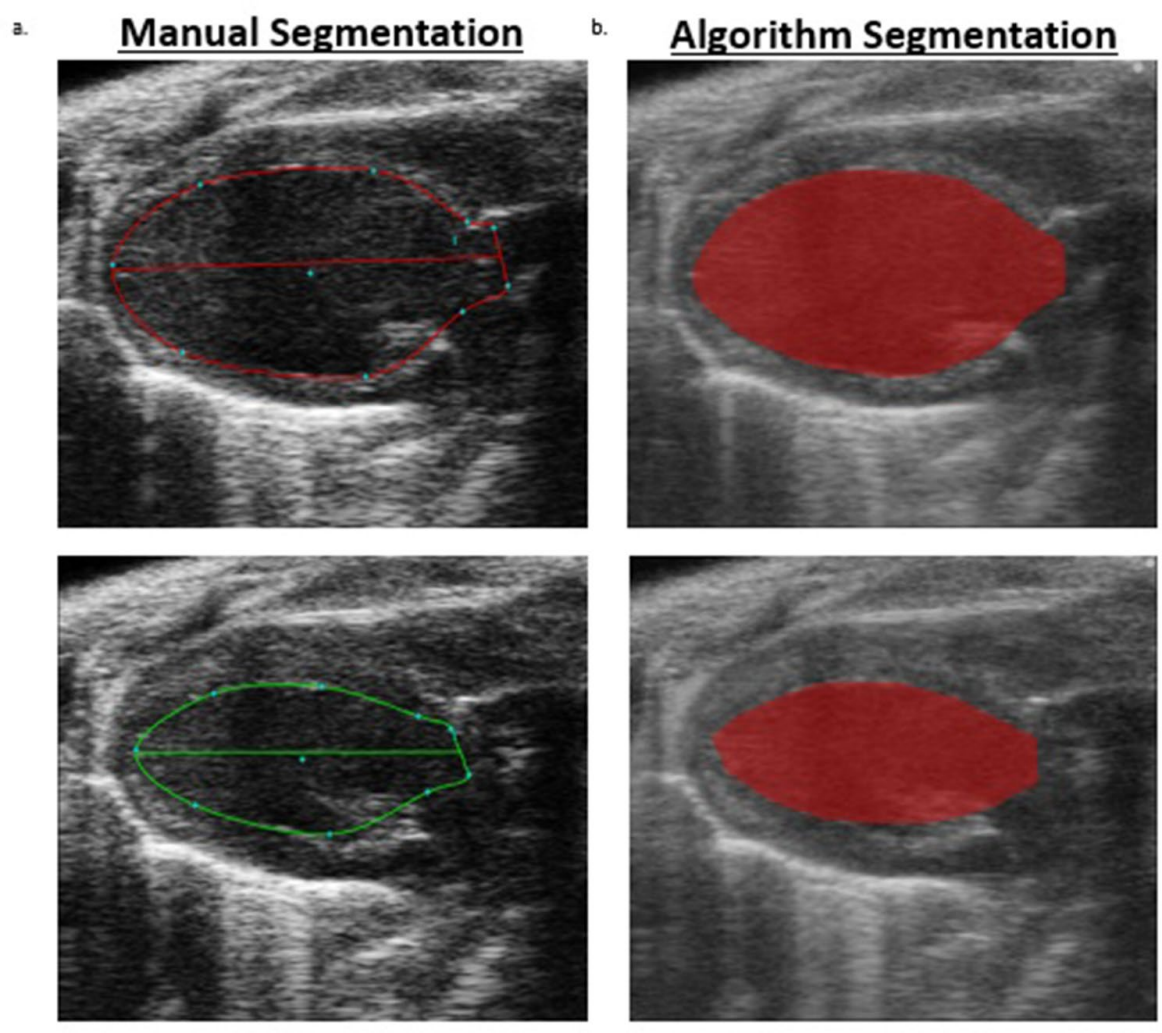
B-mode ROI’s: 2-dimensional left ventricle long axis B-mode echocardiograms from C57BL/6 mice. Visual comparison of manually segmented ROI (left) vs. ROI generated from the semi-automated segmentation algorithm of the same images (right). (**a**) shows a representative baseline long axis LV B-mode echocardiogram from a C57BL/6 mouse that was traced manually (top is the LV in end-diastole, bottom is the LV in end-systole). (**b**) Shows the same baseline LV B-mode echocardiogram, but with segmentation conducted using the semi-automated algorithm for analysis (top is LV in end-diastole, bottom is LV in end-systole).

The average coefficient of determination for dimension and volume estimations from long-axis B-mode images among the three independent sonographers analyzing one set of acquired data (Analyst 1, Analyst 2, and Analyst 3) using the algorithm for analysis compared to using the manual segmentation technique were 98.5% for end systolic area (ESA), 91.1% for end diastolic area (EDA), 98.4% for end systolic volume (ESV), and 92.7% for end diastolic volume (EDV) (Table 1a). Clinically relevant long axis functional endpoints produced average regression coefficients among the three analysts of 43.4% for fractional shortening (FS), 50.5% for stroke volume (SV), 93.3% for ejection fraction (EF), and 49.4% for cardiac output (CO) (Table 1a and Fig. 2a). Inter-user variation while using the semi-automated analysis algorithm was assessed by comparing the data sets produced from the same three individuals and performing linear regressions between each combination of users (Analyst 1 vs. Analyst 2, Analyst 3 vs. Analyst 2, and Analyst 3 vs. Analyst 1) (Table 1b and Fig. 2b). When comparing the variation in data obtained from using the algorithm between the three analysts to the variation of data obtained by manual segmentation between all three analysts, the average coefficients of determination for, ESA, EDA, ESV and EDV were 95.4%, 85.6%, 97.2%, and 91.2%, respectively, while manual segmentation produced average correlation coefficients of 96.6%, 95.4%, 96.8%, and 95.9%, respectively (Table 1b, c and Fig. 2b,c). The functional endpoints comparing between-user variation among the three analysts while using the algorithm for segmentation produced a range of coefficients of determination from 8.1% for FS, 40.5% for SV, 43.4% for CO and 92.1% for EF. In comparison, manual between-user variation produced coefficients of determination of 55.3% for FS, 71.3% for SV, 44.3% for CO and 90.8% for EF (Table 1b, c and Fig. 2b,c). Validation testing revealed that the semi-automated analysis algorithm for PLAX B-mode LV assessment correlated well to the current standard of manually tracing ultrasound images and produced comparable segmentations (Fig. 3a,b).

**Table 1:** Data tables of coefficient of determination averages (*r*^*2*^) among the three independent analysts for long axis B-mode left ventricle functional and structural end-points.

(a) B-mode_algorithm vs. manual: coefficient of determination averages among three independent analysts				
LV end point	Analyst 1	Analyst 2	Analyst 3	Average r^2^-value of three analysts (%)
End systolic area (mm^2^)	99.34	97.94	98.18	98.5
End diastolic area (mm^2^)	96.19	81.42	95.55	91.1
End systolic volume (μL)	99.51	97.80	97.89	98.4
End diastolic volume (μL)	96.97	85.68	95.42	92.7
Fractional shortening (%)	40.20	35.07	54.89	43.4
Stroke volume (μL)	81.21	7.34	63.02	50.5
Ejection fraction (%)	97.96	95.35	86.48	93.3
Cardiac output (mL/min)	71.51	32.35	44.24	49.4

(b) B-mode_algorithm vs. algorithm: coefficient of determination averages among three independent analysts				
LV end point	Analyst 1 vs. analyst 2	Analyst 3 vs. analyst 1	Analyst 3 vs. analyst 2	Average r^2^-value between three analysts (%)
End Systolic Area (mm^2^)	94.04	95.37	96.93	95.4
End diastolic area (mm^2^)	78.48	82.09	96.16	85.6
End systolic volume (μL)	96.61	96.82	98.04	97.2
End diastolic volume (μL)	86.48	90.26	96.77	91.2
Fractional shortening (%)	0.10	15.01	9.22	8.1
Stroke volume (μL)	23.82	19.86	77.86	40.5
Ejection FRACTION (%)	94.48	91.22	90.68	92.1
Cardiac output (mL/min)	45.10	22.74	62.50	43.4

(c) B-mode_manual vs. manual: coefficient of determination averages among three independent analysts				
LV end point	Analyst 1 vs. analyst 2	Analyst 3 vs. analyst 1	Analyst 3 vs. analyst 2	Average r^2^-value between three analysts (%)
End systolic area (mm^2^)	94.56	99.55	95.74	96.6
End diastolic area (mm^2^)	94.62	97.45	94.10	95.4
End systolic volume (μL)	94.92	98.76	96.68	96.8
End diastolic volume (μL)	95.75	96.89	95.15	95.9
Fractional shortening (%)	78.08	57.06	30.69	55.3
Stroke volume (μL)	79.33	62.22	72.27	71.3
Ejection fraction (%)	91.62	94.52	86.40	90.8
Cardiac output (mL/min)	68.41	28.91	35.68	44.3

(a) Compares average coefficient of determination for each analyst using the semi-automated segmentation algorithm related to using the manual segmentation technique; (b) compares within analyst coefficient of determination averages while using the semi-automated analysis algorithm; and (c) compares within analyst coefficient of determination averages when using manual segmentation. P-values were set at < 0.05.

### M-mode segmentation

**Figure 4:**
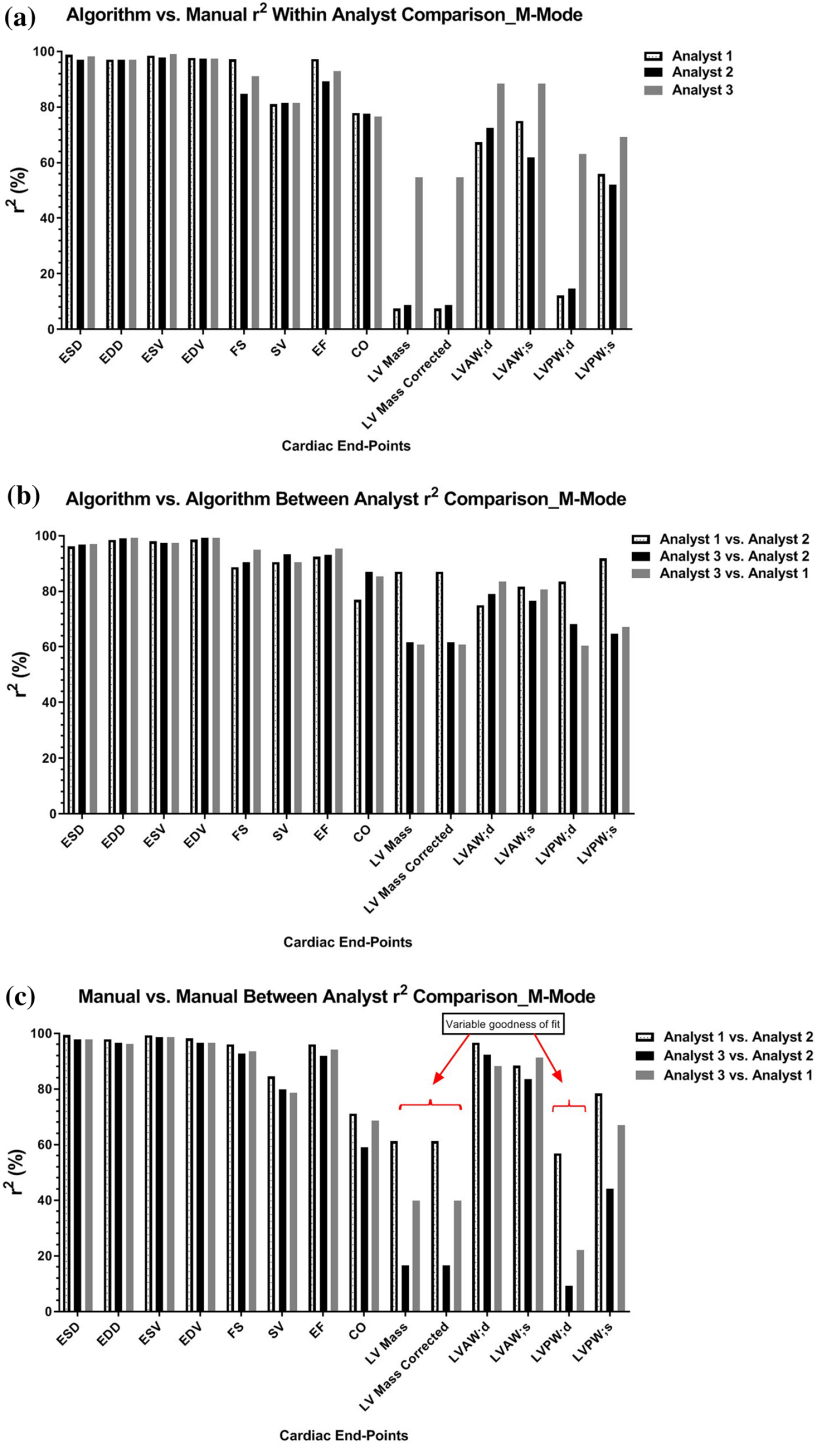
M-mode Segmentation Comparisons: Graphical representations of M-mode coefficient of determination among three analysts, with r^2^ values on the y-axis and short axis LV structure and function end-points on the x-axis. (**a**) Displays the within analyst comparison of left ventricle M-mode echocardiograms segmented using the semi-automated algorithm compared to manual segmentation. (**b**) Shows the between analyst comparison while employing the algorithm for segmentation. (**c**) Shows the between analyst comparison when manual segmentation is performed; p values set at p < 0.05. Red brackets highlight variation in structural end-points resulting in a variable goodness of fit.

**Figure 5:**
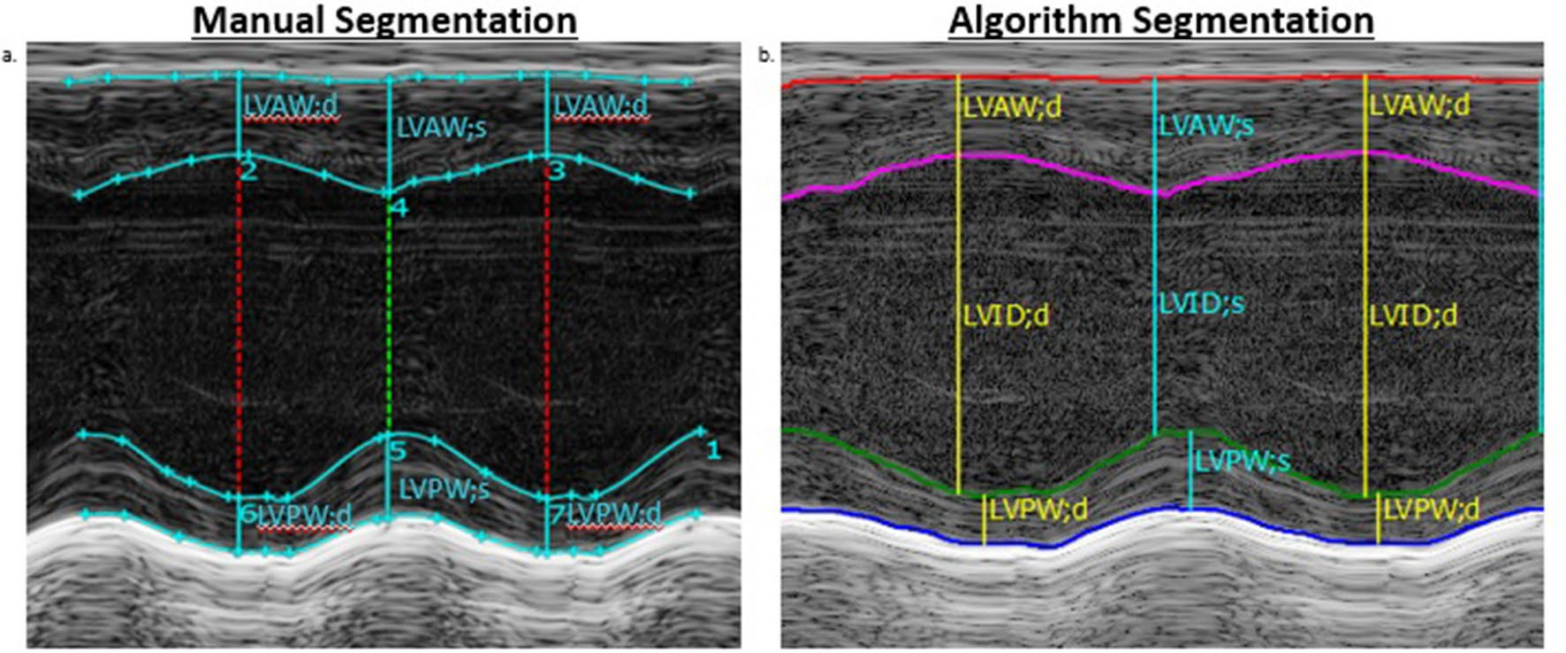
M-mode ROI’s: 2-dimensional short axis images of the left ventricle of C57BL/6 mice. Visual comparison of manually segmented ROI (left) vs. ROI generated from the semi-automated segmentation algorithm of the same images (right). (**a**) Shows a representative baseline LV M-mode echocardiogram from a mouse that was traced manually. (**b**) Shows the same baseline LV M-mode echocardiogram segmented using the semi-automated algorithm for analysis.

Head-to-head comparisons of manual- to algorithm-based data produced linear relationships with average coefficients of determination (r^2^) of 63.4% and 32.2% for systolic and diastolic posterior wall thickness measurements, and 76.0% for systolic and 75.8% for diastolic anterior wall thicknesses, respectively (Table 2a and Fig. 4a). End systolic and end diastolic dimensions and volumes produced average covariance of 98.0% for end systolic dimension, 97.4% for end diastolic dimension, 98.5% for end systolic volume, and 97.8% for end diastolic volume (Table 2a and Fig. 4a). Cardiac function measurements were also tightly correlated between both methods of analysis, with an average r^2^ of 92.3% for fractional shortening, 82.1% for stroke volume, 94.2% for ejection fraction and 77.4% for cardiac output (Table 2a and Fig. 4a). Left ventricle mass was the least concordant when comparing the two methods, with an average coefficient of determination of 25.2% (Table 2a and Fig. 4a). The between-user variation among the three analysts employing the algorithm was less than that of manual segmentation as evidenced by stronger regressions for end diastolic dimension 98.9%, end diastolic volume 99.0%, stroke volume 91.4%, cardiac output 83.1%, LV mass and LV mass corrected for body surface area 69.8%, and posterior wall thicknesses in systole 74.5% and diastole 69.8% (Table 2b and Fig. 4b) compared to the between user variation produced with manual segmentation; end diastolic dimension 96.9%, end diastolic volume 97.1%, stroke volume 80.9%, cardiac output 66.3%, LV mass corrected 39.2%, posterior wall thickness in systole 63.2% and in diastole 29.4% (Table 2c and Fig. 4c). The semi-automated analysis algorithm for SAX M-mode LV assessment produced comparable segmentations to those obtained from manual segmentation (Fig. 5a,b).

**Table 2:** Data tables of coefficient of determination averages amongst the three independent analysts for short axis M-mode left ventricle functional and structural end points.

(a) M-mode_algorithm vs. manual: coefficient of determination averages among three independent analysts				
LV end point	Analyst 1	Analyst 2	Analyst 3	Average r^2^-value of three analysts (%)
End systolic dimension (mm)	98.82	96.97	98.23	98.0
End diastolic dimension (mm)	97.05	98.19	96.92	97.4
End systolic volume (μL)	98.44	97.92	99.12	98.5
End diastolic volume (μL)	97.69	98.29	97.34	97.8
Fractional shortening (%)	97.16	88.66	91.09	92.3
Stroke volume (μL)	80.98	83.86	81.38	82.1
Ejection fraction (%)	97.29	92.37	92.83	94.2
Cardiac output (mL/min)	77.88	77.58	76.64	77.4
LV mass (mg)	7.488	13.49	54.77	25.2
LV mass corr (mg)	7.488	13.49	54.77	25.2
LVAW; d (mm)	67.38	71.62	88.45	75.8
LVAW; s (mm)	75.03	64.46	88.43	76.0
LVPW; d (mm)	12.16	21.33	63.13	32.2
LVPW; s (mm)	55.88	64.91	69.26	63.4

(b) M-mode_algorithm vs. algorithm: coefficient of determination averages among three independent analysts				
LV end point	Analyst 1 vs. analyst 2	Analyst 3 vs. analyst 1	Analyst 3 vs. analyst 2	Average r^2^-value between three analysts (%)
End systolic dimension (mm)	96.11	96.91	96.77	96.6
End diastolic dimension (mm)	98.51	99.15	99.01	98.9
End systolic volume (μL)	97.99	97.31	97.49	97.6
End diastolic volume (μL)	98.61	99.28	99.24	99.0
Fractional shortening (%)	88.63	94.95	90.37	91.3
Stroke volume (μL)	90.46	90.36	93.37	91.4
Ejection fraction (%)	92.45	95.37	93.02	93.6
Cardiac output (mL/min)	76.97	85.33	86.89	83.1
LV mass (mg)	87.00	60.89	61.58	69.8
LV mass corr (mg)	87.00	60.89	61.58	69.8
LVAW; d (mm)	74.96	83.61	78.91	79.2
LVAW; s (mm)	81.62	80.65	76.65	79.6
LVPW; d (mm)	87.00	60.89	61.58	69.8
LVPW; s (mm)	91.86	67.12	64.66	74.5

(c) M-mode_manual vs. manual: coefficient of determination averages among three independent analysts				
LV end point	Analyst 1 vs. analyst 2	Analyst 3 vs. analyst 1	Analyst 3 vs. analyst 2	Average r^2^-value between three analysts (%)
End systolic dimension (mm)	99.4	97.84	97.75	98.3
End diastolic dimension (mm)	97.82	96.17	96.62	96.9
End systolic volume (μL)	99.24	98.61	98.62	98.8
End diastolic volume (μL)	98.17	96.58	96.52	97.1
Fractional shortening (%)	96.06	93.59	92.63	94.1
Stroke volume (μL)	84.45	78.58	79.65	80.9
Ejection fraction (%)	96.01	94.12	91.79	94.0
Cardiac output (mL/min)	71.13	68.71	59.08	66.3
LV mass (mg)	61.19	39.75	16.6	39.2
LV mass corr (mg)	61.19	39.75	16.6	39.2
LVAW; d (mm)	96.62	88.19	92.2	92.3
LVAW; s (mm)	88.41	91.29	83.52	87.7
LVPW; d (mm)	56.85	22.16	9.3	29.4
LVPW; s (mm)	78.32	66.96	44.21	63.2

(a) Compares average coefficient of determination for each analyst using the semi-automated segmentation algorithm compared to using the manual segmentation technique. (b) Compares within analyst coefficient of determination averages while using the semi-automated analysis algorithm. (c) Compares within analyst coefficient of determination averages when using manual segmentation.

### Coefficients of variation

**Figure 6:**
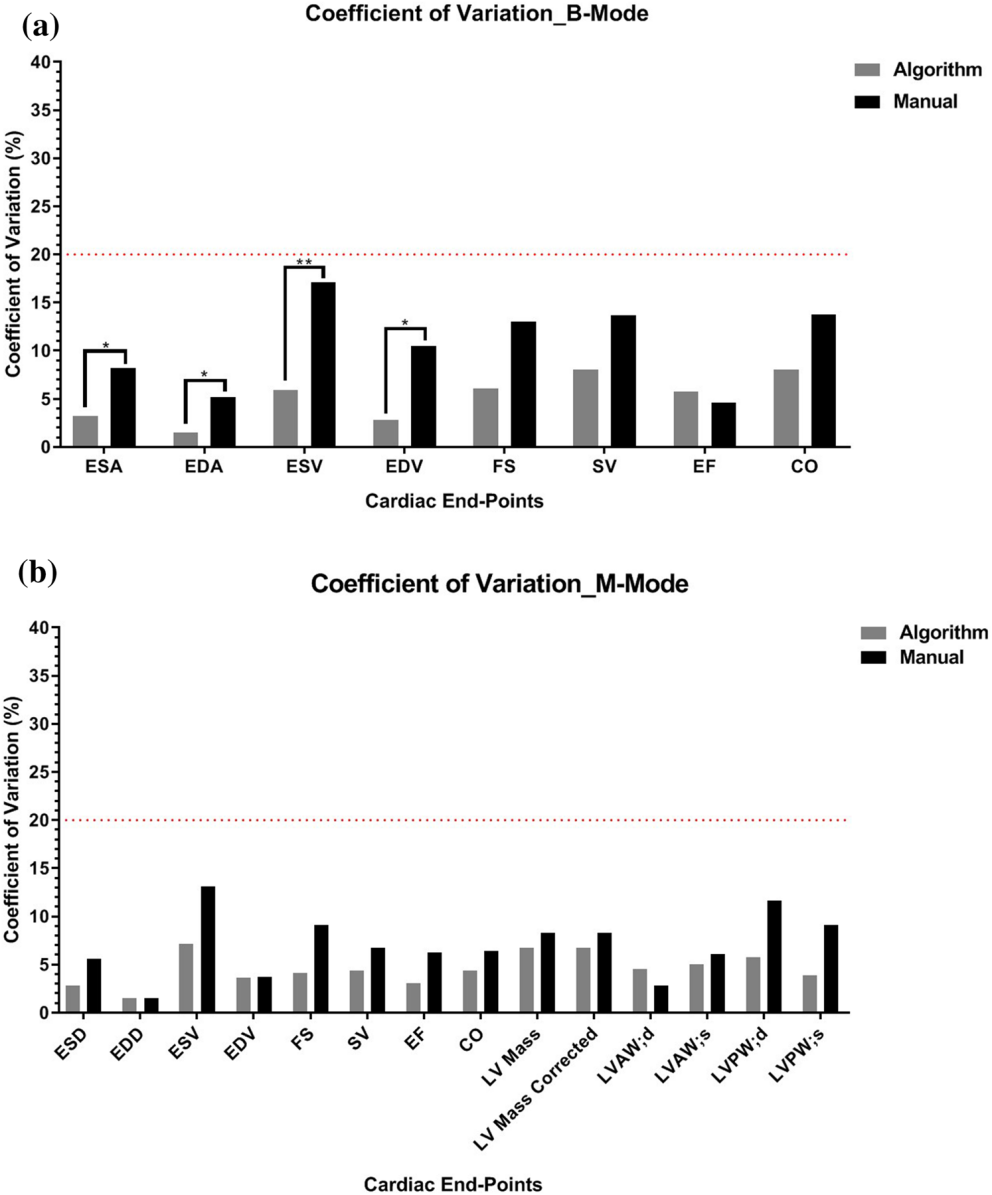
Analysis of variation: coefficient of variation analysis displaying coefficient of variation percentage on the y-axis and LV structural and functional end-points on the x-axis. Dark gray bars represent analysis performed using the semi-automated algorithm and black bars represent use of manual segmentation. Red dotted line shows internally set limit of variation percentages. (**a**) Details variation observed with long axis B-mode evaluation. (**b**) Details variation observed with short axis M-mode evaluation. Student within-subject t-tests were done on each cardiac end-point; *p < 0.05; **p < 0.01.

Coefficient of variation analysis was also performed using one sonographer to measure the same set of images in triplicate. These procedures were followed for manual segmentation as well as for segmentation with the semi-automated algorithm for both long- and short-axis. This was done to assess the reproducibility of the method and to evaluate how it compares to the standard manual segmentation approach. Variation of long-axis B-mode structural and functional endpoints improved by ~ 2–11% in the data set analyzed using the semi-automated segmentation tool (Table 3a and Fig. 6a). Repeated analysis of short-axis M-mode measurements of left ventricular structure and function displayed ~ 1–6% less variation employing the semi-automated segmentation tool as compared to manual analysis (Table 3b and Fig. 6b).

**Table 3:** Data tables displaying coefficient of variation analysis percentages between manual segmentation and semi-automated algorithm segmentation.

(a): B-mode coefficient of variation manual vs. algorithm		
LV end point	Manual (%)	Algorithm (%)
End systolic area (mm^2^)	8.2	3.2
End diastolic area (mm^2^)	5.2	1.5
End systolic volume (μL)	17.1	5.9
End diastolic volume (μL)	10.5	2.8
Fractional shortening (%)	13.0	10.3
Stroke volume (μL)	13.7	8.0
Ejection fraction (%)	4.6	5.8
Cardiac output (mL/min)	13.8	8.0

(b): M-mode coefficient of variation manual vs. algorithm		
LV end point	Manual (%)	Algorithm (%)
End systolic dimension (mm)	5.6	3.5
End diastolic dimension (mm)	1.5	1.5
End systolic volume (μL)	13.1	8.9
End diastolic volume (μL)	3.8	3.7
Fractional shortening (%)	9.1	4.8
Stroke volume (μL)	6.7	3.6
Ejection fraction (%)	6.3	3.6
Cardiac output (mL/min)	6.4	4.4
LV mass (mg)	8.3	7.0
LV mass corr (mg)	8.3	7.0
LVAW; d (mm)	2.8	5.0
LVAW; s (mm)	6.1	5.6
LVPW; d (mm)	11.7	4.5
LVPW; s (mm)	9.1	3.5

(a) details long axis B-mode COV averages and (b) details short axis M-mode COV averages from analyzing the same data set of echocardiograms in triplicate using the manual analysis method compared to using the semi-automated segmentation algorithm.

## Discussion

A key advantage of echocardiography is that it presents as a non-invasive, in vivo imaging modality allowing for the longitudinal characterization of cardiovascular disease progression, in both preclinical and clinical practice. For murine pharmacology studies, serial micro-ultrasound acquisition produces an extraordinary amount of data and requires many hours of analysis and data processing from a highly trained sonographer to generate decision making data. The primary objective of this work was to develop a software-based analysis algorithm to address the data bottleneck that preclinical murine ultrasound image analysis presents in the drug discovery and development arena. The tool that was described herein addressed the inherent limitations experienced with ultrasound data analysis industry wide. As demonstrated by the high degree of reproducibility and noted reduction in user time required to analyze, we successfully created and deployed a semi-automated analysis algorithm for the derivation of LV PLAX B-mode and SAX M-mode volumetric and functional parameters for murine echocardiograms.

The tool was rigorously validated and measured against the current method of manual segmentation. Isoproterenol is a synthetic and potent beta-adrenergic agonist with known cardiac stimulating properties including inducing LV hyper-contractility^14^. The robust, measurable response observed with Isoproterenol facilitated testing of the functionality and boundaries of the semi-automated segmentation tool under pharmacologic challenge, with the intent of identifying any limitations. The tightly correlated values seen in ESA, EDA, ESV and EDV (Table 1a and Fig. 2a) were expected when you consider the principles behind the PLAX B-mode algorithm and that it functions mainly on shape and size of the region of interest. Functional data point correlations are not as tight between methods due to slight variations within the equation used to calculate them and these differences in calculated volumetric endpoints become amplified once further manipulated to obtain values of ejection fraction and stroke volume. The modified Simpson’s rule, also known as the biplane method of disks, is the recommended method of choice by the American Society of Echocardiography (ASE) committee for 2D LV volumetric measurements^15–17^.

$$V=({A}_{m}\text{)}\frac{L}{3}\text{+ (}\frac{{A}_{m}+{A}_{p}}{2}\text{ )}\frac{L}{3}\text{ + }\frac{I}{3}\text{(}{A}_{p}\text{)}\frac{L}{3}$$

The underlying concept is that total LV volume is calculated from the aggregate of a predetermined number of elliptical disks of segmented volumes along the longitudinal axis of the LV chamber^15^.
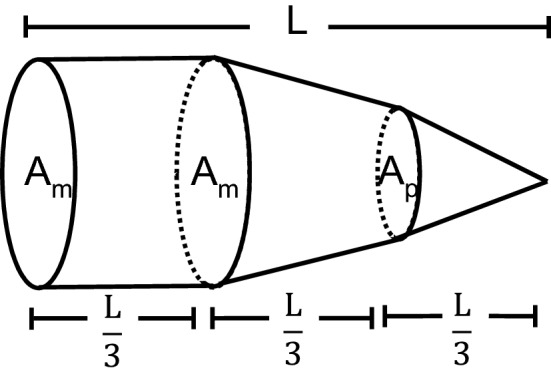


This method is advantageous because it corrects for shape distortions within the LV and has less geometric assumptions compared to other methods that rely heavily on linear dimensions^16^. The B-mode semi-automated segmentation algorithm and the Visualsonics software for manual segmentation both employ the modified Simpson’s rule in relation to their mathematical assumptions of volume, but may differ in the number of disks used within the equation^15^. ASE recommends the number of predetermined segmented disks to be between one and twenty, with twenty being what is typically used^17^. For that reason, the semi-automated algorithm was developed to calculate total LV volume based on the summation of twenty disks, whereas the number of disks used within the software for manual segmentation is unknown. Therefore, variation in the number of disks used for calculations of ESV and EDV could be contributing to the larger differences seen in functional endpoints between the two segmentation methods. Consequently, the semi-automated algorithm did not reduce the intra-user variation among the three sonographers for PLAX assessments when compared to manual intra-user variation except for endpoints of ESV and EF (Table 1b, c and Fig. 2b,c). Coefficient of variation analysis displayed that one can obtain more consistent data with a single user employing the algorithm than with manual segmentation (Table 3a, b and Fig. 6a,b). To validate that the system is reproducible within itself, further testing was performed. The same data set was run three separate times, while picking the exact same diastolic and systolic frames for long axis B-mode images. No variation in ROI’s was observed when comparing the system against itself in a repeat-analysis fashion (data not shown). The consistency observed in ROI’s generated by the algorithm when selecting the same frames throughout each iteration of measurements verifies that there are no underlying discrepancies within the algorithm script that would cause erroneous ROI annotation.

When comparing short-axis M-mode data obtained from the three sonographers using both methods of segmentation, the results were generally tightly correlated, suggesting good agreement between manual- and algorithm-based analyses of left ventricle echocardiograms (Fig. 4a). The low r^2^ values represented in the structural endpoints of LV Mass and LV Mass corrected for body surface area (Fig. 4a) likely are the result of variations observed in manual LV wall thickness analysis, as is evident in the manual vs. manual comparison (Fig. 4c). The algorithm approach also worked to decrease the intra- user variation which is a major concern surrounding the utility of ultrasound technology. The algorithm automatically populates dimension lines for anterior and posterior wall thicknesses within the short-axis M-mode segmentation. This eliminates the subjective variation of individual analysts determining line placements by eye. Therefore, this approach can be used by multiple analysts and produce similar results among them, compared to the current standard of manual segmentation. Coefficient of variation analysis showed that the algorithm performed better at decreasing data variation than manual analysis in almost every long- and short-axis endpoint and statistically better for end points of systolic area, systolic volume, diastolic area and diastolic volume (Fig. 6a,b). The semi-automated algorithm is reproducible between different analysts as well as within one analyst (Figs. 4b, 6a,b).

**Figure 7:**
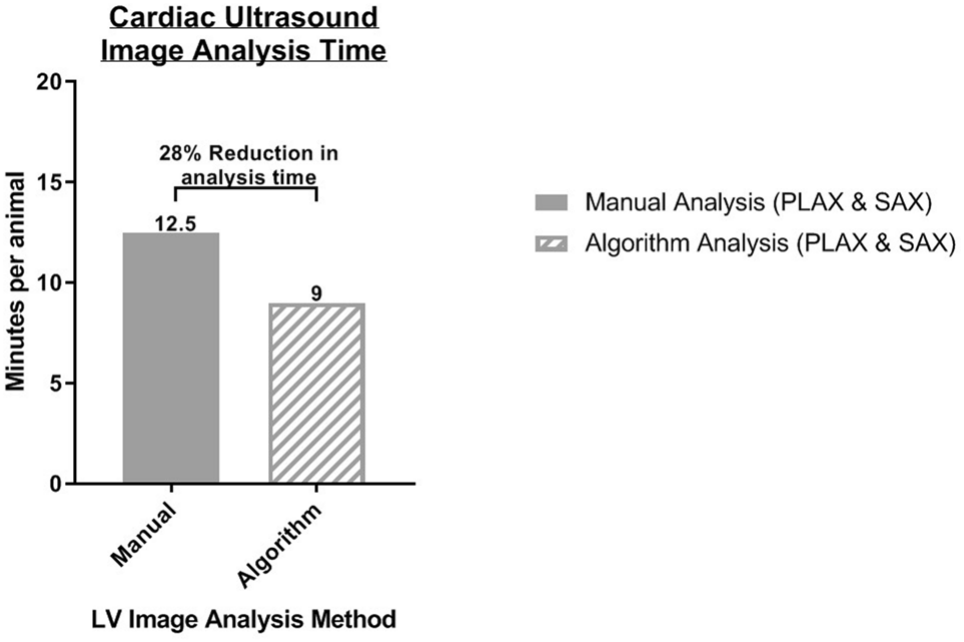
Time of analysis comparison: graphical representation of time required for left ventricle ultrasound image analysis using a manual segmentation method compared to using the semi-automated segmentation algorithm tool.

The algorithm also showed great accuracy in its ability to point out the same outliers that were seen with manual segmentation. Being able to detect these differences are essential in phenotypic characterization and preclinical drug development. The inherent low signal to noise that accompanies ultrasound images compared to other imaging modalities makes manual analysis a tedious, repetitive behavior often accompanied by physical eye strain of the analyzer (personal experience and anecdotal evidence shared by peers). In contrast to manually placing individual points along the endo- and epicardium for diastolic and systolic phases for every animal within a study, the semi-automated algorithm approach allows for hands-free initial segmentation and overnight batch processing. In turn, this permits an analyst to multi-task and even analyze multiple studies at the same time, substantially cutting down analysis time and expediting data processing. After validation testing was complete, a second internal audit of time required to analyze murine echocardiograms was performed and proved that the semi-automated analysis algorithm reduces the time required for PLAX and SAX image analysis by 28% (Fig. 7). Reducing image analysis time in combination with the capability to work on multiple studies at once will greatly decrease the bottleneck associated with micro-ultrasound data processing.

The semi-automated segmentation algorithm performs very well in comparison to manual analysis but does present with several limitations. Images that are acquired without clear visualization of essential landmarks and with less defined endocardial walls for PLAX are difficult for a trained human to analyze, and therefore pose the same challenge for a trained computer, culminating in a less accurate ROI annotation. Another challenge we encountered was seen in the reporting of endpoints such as heart rate, temperature and cardiac output. In order to have the algorithm report temperature and heart rate, the latter being an essential component for calculation of cardiac output, the sonographer needs to specify the acquisition protocol within the ultrasound scanning system itself before the time of acquisition. If the heart rate data is not populated within the header of the dicom files, the algorithm and iPACS will not be able to generate these endpoints in the subsequent data sheet. We hope to develop the algorithm further to incorporate automatic calculations of heart rate and cardiac output from the chamber dimensions of each cardiac phase in the M-mode images as an alternative. A final limitation is that although the tool is straight forward in functionality and generates high quality, reliable and reproducible data, it still requires training and human intervention. Future work will continue to develop this segmentation method, incorporating aspects of artificial intelligence and machine learning to further make the tool more accurate for long-axis measurements, as well as remove the manual intervention of selecting end systolic and end diastolic frames for each animal. Producing more accurate long-axis ROI's will also work to decrease the variability seen with long-axis functional endpoints.

Machine learning and algorithm-based automation is well-suited for the concept of pattern recognition present in preclinical echocardiogram image analysis^18^. Preclinical cardiovascular ultrasound studies produce very large volumes of data. The manually intensive process of storing, transferring, analyzing, and aggregating this data is time consuming and costly. Reducing many of these repetitive tasks through workflow automation improves productivity, reduces both image analysis time and subjective bias, facilitating rapid progression through studies. These streamlined methods can be applied to not only other ultrasound modalities used in cardiovascular research such as pulse-wave Doppler for vascular blood flow assessments but other preclinical applications including tumor volume measurements for oncology research.

## Conclusion

Clinical ultrasound technology is critical for diagnosis of cardiovascular disease, monitoring disease progression, and assessment of therapeutic interventions. The quantitative endpoints used in clinical practice can similarly be assessed in rodents, making these data vitally important for go/no-go decisions in the preclinical drug development process. The inherent challenges of the time required for ultrasound image analysis and the inter-observer data reproducibility experienced industry wide were the motivation behind the creation of this semi-automated segmentation workflow. The data we have presented demonstrate that a semi-automated segmentation tool can provide the ability to rapidly analyze data with a degree of accuracy between multiple observers that is superior to current manual approaches. Creating a semi-automated workflow eliminates the time consuming and repetitive characteristics of ultrasound analysis, decreases overall image analysis time, produces reproducible data and reduces the inter- and intra-operator variability by limiting subjective biases. This promises to facilitate the generation of data and expedite critical decisions facilitating appropriate movement of therapeutic programs.

## References

[1] James ML, Gambhir SS (2012). A molecular imaging primer: Modalities, imaging agents, and applications. Physiol. Rev..

[2] Foster FS, Zhang MY, Zhou YQ (2002). A new ultrasound instrument for in vivo microimaging of mice. Ultrasound Med. Biol..

[3] DeGroff CG (2002). Doppler echocardiography. Pediatr. Cardiol..

[4] Hoit BD, Walsh RA (1997). In vivo echocardiographic assessment of left ventricular function in transgenic and gene-targeted mice. Trends Cardiovasc. Med..

[5] Scherrer-Crosbie M, Thibault HB (2008). Echocardiography in translational research: Of mice and men. J. Am. Soc. Echocardiogr..

[6] Pistner A, Belmonte S, Coulthard T (2010). Murine echocardiography and ultrasound imaging. J. Vis. Exp..

[7] Ram R, Mickelsen DM, Theodoropoulos C (2011). New approaches in small animal echocardiography: Imaging the sounds of silence. Am. J. Physiol. Heart Circ. Physiol..

[8] Nobel JA (2016). Reflections on ultrasound image analysis. J. Med. Image Anal..

[9] Soa P, Hegadi R, Karmakar S (2014). A literature review on approaches of ECG pattern recognition. Int. J. Inf. Sci. Intell. Syst..

[10] Vivoquant: Invicro. https://www.invicro.com/vivoquant-home/. (2018).

[11] Statistical Parametric Mapping: An Overview. https://www.sciencedirect.com/topics/medicine-and-dentistry/statistical-parametric-mapping. (2018).

[12] Dynamic programming: Algorithm. https://en.wikipedia.org/wiki/Dynamic_programming. (2018).

[13] iPACS: Invicro. https://www.invicro.com/ipacs/. (2018).

[14] Isoprenaline|C11H17NO3: PubChem https://pubchem.ncbi.nlm.nih.gov/compound/isoproterenol. (2018).

[15] Fondall ED, Parisi AF, Moynihan PF (1979). Assessment of left ventricular ejection fraction and volumes by real-time, two-dimensional echocardiography: A comparison of cineangiographic and radionuclide techniques. Circulation.

[16] Kosaraju, A., Makaryus, A.N. Left Ventricular Ejection Fraction. *NCBI Bookshelf. A service of the National Library of Medicine, National Institutes of Health. [Bookshelf ID: NBK459131] [PubMed: 29083812].* (2019).

[17] Lang RM, Badano LP, Mor-Avi V (2015). Recommendations for cardiac chamber quantification by echocardiography in adults: An update from The American Society of Echocardiography and The European Association of Cardiovascular Imaging. J. Am. Soc. Echocardiogr..

[18] Maraci MA, Bridge CP, Napolitano R (2017). A framework for analysis of linear ultrasound videos to detect fetal presentation and heartbeat. J. Med. Image Anal..

